# Estimating sediment yield at Kaduna watershed, Nigeria using soil and water assessment tool (SWAT) model

**DOI:** 10.1016/j.heliyon.2019.e02106

**Published:** 2019-07-19

**Authors:** J. Daramola, T.M. Ekhwan, J. Mokhtar, K.C. Lam, G.A. Adeogun

**Affiliations:** a Geography Program, Social, Environmental, Development, Sustainability Research Centre (SEEDS), Faculty of Social Sciences and Humanities, Universiti Kebangsaan Malaysia, 43600, Bangi, Selangor, Malaysia; b Department of Civil Engineering, Kwara State University, P.M. B 1530, Molete, Kwara State, Nigeria

**Keywords:** Environmental management, Sustainable development, Physical geography, Remote sensing, Hydrology, SWAT, Sediment yield, Soil erosion, Sustainability, Reservoir, Kaduna watershed

## Abstract

Over the years, sedimentation has posed a great danger to the storage capacity of hydropower reservoirs. Good understanding of the transport system and hydrological processes in the dam is very crucial to its sustainability. Under optimal functionality, the Shiroro dam in Northern Nigeria can generate ∼600 MW, which is ideally sufficient to power about 404,000 household. Unfortunately, there have not been reliable monitoring measures to assess yield in the upstream, where sediments are sourced into the dam. In this study, we applied the Soil and Water Assessment Tool (SWAT) to predict the hydrological processes, the sediment transport mechanism and sediment yield between 1990 and 2018 in Kaduna watershed (32,124 km^2^) located upstream of the dam. The model was calibrated and validated using observed flow and suspended sediment concentration (SSC) data. Performance evaluation of the model was achieved statistically using Nash-Sutcliffe (NS), coefficient of determination (r^2^) and percentage of observed data (p-factor). SWAT model evaluation using NS (0.71), r^2^ (0.80) and p-factors of 0.86 suggests that the model performed satisfactorily for streamflow and sediment yield predictions. The model identified the threshold depth of water (GWQMN.gw) and base flow (ALPHA_BF.gw) as the most sensitive parameters for streamflow and sediment yield estimation in the watershed. Our finding showed that an estimated suspended sediment yield of about 84.1 t/ha/yr was deposited within the period under study. Basins 67, 71 and 62 have erosion prone area with the highest sediment values of 79.4, 75.1 and 73.8 t/h respectively. Best management practice is highly recommended for the dam sustainability, because of the proximity of erosion-prone basins to the dam.

## 1 Introduction

The greatest challenge of dam construction is a reduction in storage capacity due to reservoir sedimentation (WCD, 2000; Gottschalk, 1964; Petts, 1984; Cogollo and Villela, 1988; Evans et al., 2000). Studies have shown that sediment yield into reservoir poses a great danger to dam globally (SedNet, 2006; Syvitsky et al., 2005; Ezugwu, 2013; Palmieria et al., 2001; De Cesare et al., 2001). Reservoir sedimentation, according to Morris and Fan (1998) is the process by which stream transports and deposits sediments in the reservoir behind the dam. This suggests that the reservoirs formed on the natural course of the river are subject to some level of sediment influx and deposition (Sheikh and Aliyu, 2017; Toriman et al., 2012; Julien, 2010; Zhide and Yuqian, 2003). Sediments in reservoirs constitute problems for both physical and chemical characteristics of the water quality (Alemayehu et al., 2014). Physically, sediments affect the useful life of reservoirs as well as damage the aesthetic quality of the environment. Chemically, it serves as a distribution medium for certain toxins and a source for the overlying water column and biota within a reservoir (Juracek and Stiles, 2010; Alemayehu et al., 2014). According to Reza (2011), reservoir sedimentation increases the risk of water runoff during floods, reduces the quality of consumed water, decreases reservoir water volume and decreases the dam's life cycle.

Although realistic datasets are an essential part of dam management practices, Nigeria dams generally lack suitable data due to poor tradition on the research of dams (Sheikh and Aliyu, 2017). Only a few independent researchers have executed studies on dam monitoring in Nigeria. Adeogun et al. (2015), studied sediment yield at the upstream watershed of Jebba lake using SWAT Mapwindow and predicted estimated sediment yield of 255.8 tons/ha/yr. However, this forecast is considered too high for north-central Nigeria, taking the climatic conditions of the region into consideration. Adediji and Fashae (2014) determined suspended sediment concentration in river Awba catchment, Ibadan south-west Nigeria using laboratory filtration method. Tukur et al. (2013) studied sedimentation in the transportation of canals in the Kano River Irrigation project, North-west Nigeria using volumetric analysis and filtration methods. The methods adopted by these authors are tedious, time-consuming, and require a high analytical cost and energy, especially when dealing with the large watershed area.

Abam (2001) established that River Kaduna, where the Shiroro dam (the study area) is located, receives sediment influx of about 96 t/km^2^ (66.6 × 10^6^) per year. Adeogun et al. (2018) validated this fact that information collected on the three Nigeria hydropower reservoirs (Shiroro, Kanji and Jebba) confirmed that the storage capacities of these dams have been seriously affected by considerable movement and deposition of sediment from upstream of the dams to the reservoirs. These have led to negative impacts such as persistent flood, reduction in the reservoir life and benefits in the area of power generation, irrigation, water supply, flood control, navigation, wildlife development, recreation, sanitation, and groundwater recharge (Ezugwu, 2013).

Sedimentation in Jebba dam according to Adeogun et al. (2018) accounted for 3,177 MW loss in power generation within 14 years. The decrease in hydropower energy generation in Nigeria dams has resulted in persistent power failure and rationing. The nation's actual daily power generation fell to less than 2,000 megawatts (MW) in the year 1999 and generation went down from an installed capacity of about 5,200 to 1,750 megawatts (MW), as compared to a load demand of 6,000 megawatts (Daily Trust, 2007). This, according to Gana and Amodu (2008) is unreliable and unable to meet the demands placed on it. Ekop (2005) affirmed that large gaps exist between demand and supply for domestic, commercial, and industrial uses, while frequent power failure and rationing is a daily occurrence in Nigeria. Public electricity in Nigeria at 6,500 MW installed capacity is at best 30% of current requirements and averagely most homes have access to the public electricity supply for six hours daily (Awofeso, 2011). These situations today have not witnessed any improvement because some villages and towns in Nigeria may not see public electricity for days. Hence, sediments' model analysis result can be a good starting point for Nigeria watershed databank upon which government, planners and dam managers can build. It can also serve as a base for watershed databank, upon which subsequent studies could be built within the study area at a watershed scale. These predictions make the study suitable as a management tool for planning and decision making in Kaduna watershed, Nigeria.

Hence, the need for a hydrological model that can address this problem in the country's watershed is urgent. The issue of sediment yield modeling has fascinated and gained the attention of many scientists but the problems of resources and convincing methods to predict sediment yields are some of the holdups towards this course. Several empirical models based on geomorphologic parameters have been established for the watershed valuation of soil erosion and sediment yield (Mishra et al., 2015). However, among these models SWAT has been identified by scholars to have considerable repute as a model to quantify the impact of land management practices in large complex watersheds, and it has been used all over the world including many developing countries and the USA where it originated (Williams et al., 2008; Neitsch et al., 2005; Spruill et al., 2000). In this study, we present the first detailed characterization of the hydrological process and sedimentation mechanisms of the Shiroro dam upstream, northern Nigeria, with a view to predicting sediment loads into basins; thus, providing clues on sediment influx into the dam as well as areas prone to erosion.

### 1.1 Location and description of the study area

**Fig. 1:**
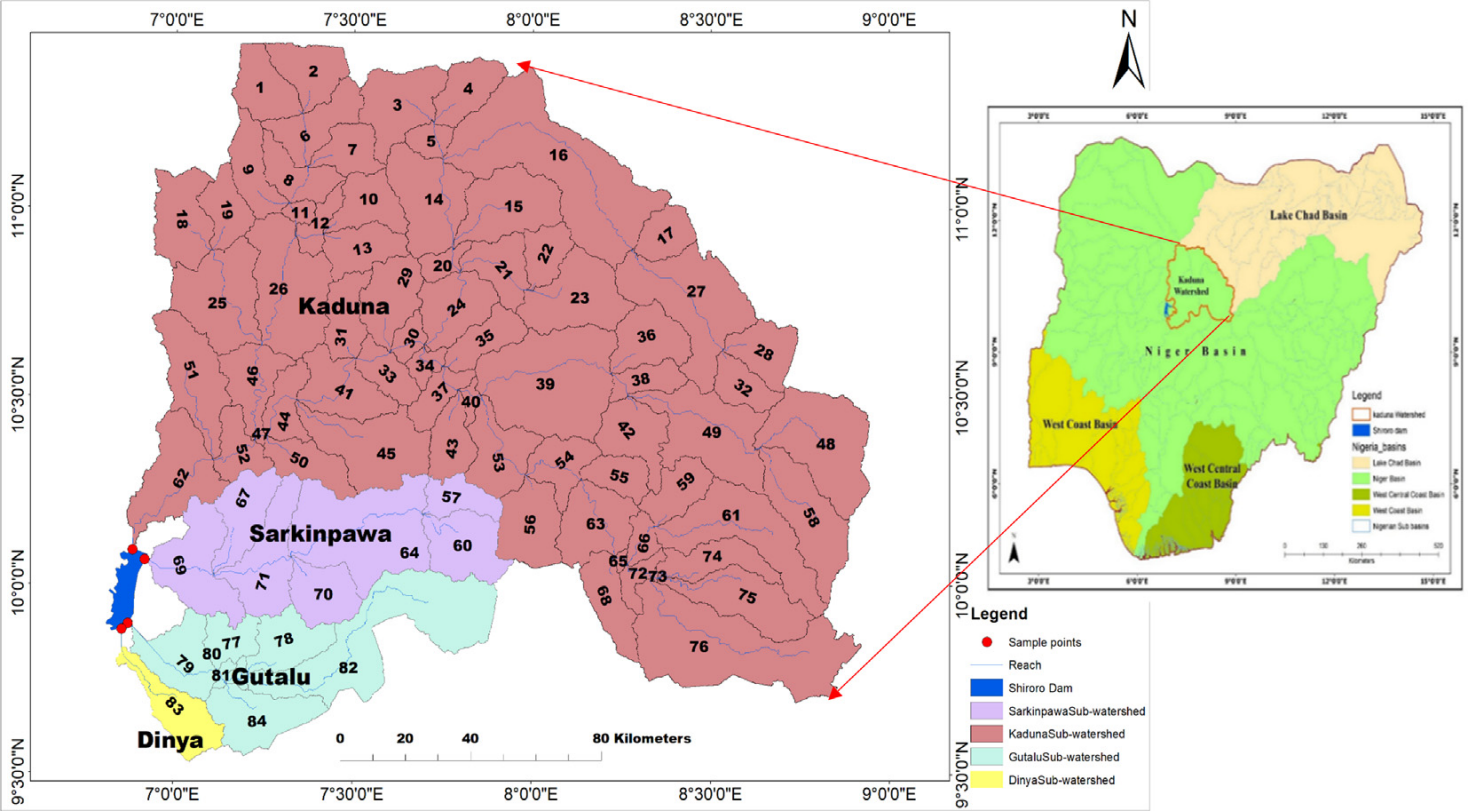
Major Sub-Watershed, Reaches and sample points of Kaduna Watershed (Inset Nigeria Map).

The study area is upstream Shiroro dam north-central Nigeria, West Africa, located between Latitude 9.35°N and 11.28°N and Longitude 6.45°E and 8.55°E with an estimated land area of 32,125 km^2^. The elevation range of the watershed is between 377 m and 1,544 m and with a mean elevation of 683 m above sea level. The Kaduna watershed (32,125 km^2^) comprises of four sub-watersheds named after the four major rivers (Kaduna, Sarkinpawa, Gutalu and Dinya). The Kaduna sub-watershed (25,675km^2^) consists of 69 basins, Sarkinpawa (3,413km^2^) and, Gutalu (2,672 km^2^) consists of seven basins each while Dinya (365km^2^) consists of one basin (Fig. 1). The four rivers and their tributaries constitute the sources of water supply to Shiroro reservoir (Fig. 1).

The geology of the study area is part of the basement complex made up of pre-Cambrian rocks that were formed over 1,500 million years ago and regarded as the oldest and most stable rock in Nigeria (Areola et al., 2014). It contains old igneous rock and metamorphic rocks like granites, migmatites and remnants of ancient sedimentary rock series, already subjected to weathering to form quartzite, schist, marble etc. The soil in the study area is predominantly sandy loam soil which constitutes about 41.94% of the total soil. The watershed vegetation consists of Guinea and Sudan savannah, which is mainly characterized by tall grasses and scattered trees. Economic activities of the people living within the watershed are farming (subsistence farming), fishing, hunting, trading and weaving.

The region experiences single maximum rainfall that lasts for about six months, from April to September followed by a dry season. The mean annual rainfall declines from 1,200 mm in the south to about 1,000 mm in the north (Areola et al., 2014); hence, the northern part of the watershed is a little drier than the south. The sedimentary geology nature of the study area alongside the predominately sandy soil with Guinea and Sudan savannah vegetation cover makes the study area susceptible to soil erosion which is a major sediment yield driver.

## 2 Material and method

Model input data includes Digital Elevation Model (DEM), of 30 m resolution from Shuttle Radar Topography Mission (SRTM), land cover classification map of West Africa of 2 km resolution obtained from U.S. Geological Survey Earth Resources Observation and Science (USGS EROS), and the 1 km resolution Soil map of Nigeria (soil types and texture), extracted from the FAO Soil database (2009). Daily weather data including, precipitation, minimum and maximum temperature, relative humidity, wind and solar radiation were obtained from two stations, Shiroro dam metrological station and Nigeria Metrological Station (NIMET). For detail information on SWAT model see (Winchell et al., 2010; Arnold et al., 2012a,b; Neitseh et al., 2009).

The streamflow data of the four reaches that constituted Kaduna watershed were obtained from the African Flood and Drought Monitor developed by Princeton Climate Analytics (PCA). Suspended sediment sampling was carried out for a period of eight-months along the four major reaches that supply water to the reservoir from where suspended sediment concentration (SSC) and turbidity were measured. The water samples were limited to four points close to the reservoir due to security challenges up north and central parts of the watershed.

The model was parameterized using the SWAT basic hydrological groups; land use, soil texture and sub-basin number (Abbaspour, 2015).

The sensitivity analysis was based on the watershed parametrization results. The observed monthly streamflow hydrological data of the four reaches that transverses the watershed was used for spatial calibration and validation of the model parameters. Literatures recommends that streamflow, sediment and nutrients transport be sequentially calibrated (Santhi et al., 2001; Engel et al., 2007), due to shared transport processes as a result of constituent interdependencies (Arnold et al., 2012a,b). SWAT-CUPSUFI2 was used for the spatial calibration and validation processes via the observed stream flow of basins 62 and 79 of 2015–2017 and sediment concentration of basins 69 and 83 of March–October 2018 (Fig. 1). The calibrated data was validated using the observed streamflow of basins 69 and 83 and sediment concentration of basins 62 and 79 of March–October to 2018 because of their similarity in climatic, soil and land use data (Eckhardt and Arnold, 2001; van Liew and Garbrecht, 2003; Cao et al., 2006; Parajuli et al., 2009). The model performance was evaluated using Nash-Sutcliffe (NS), Coefficient of determination (r^2^) and Percentage of observed data (p-factor) (Arnold et al., 2012a,b).

## 3 Results and discussion

### 3.1 The sensitivity of sediment parameters to model output

A total of 16 model parameters were evaluated to determine their relative Sensitivity to flow in the watershed. The result shows that five of the parameters are the most sensitive with less than 0.55 P values and higher t stat values of between 0 and 2.5. The flow sensitive hydrological modelling parameters for the studied watershed starting with the most sensitive are threshold depth of water in the shallow aquifer required for return flow to occur mm H_2_O (GWQMN.gw), effective hydraulic conductivity in main channel alluvium mm/hr (CH_K2.rte), soil available water capacity (SOL_AWC(.).sol), plant uptake compensation factor (EPCO.hru) and surface runoff lag coefficient (SURLAG.bsn).

Global sensitivity analysis for sediment parameters was carried out using the streamflow validation parameters and values as a base. Added to these are other sediment parameters such as SPEXP.bsn, SPCON.bsn, SOL_BD(..).sol, OV_N.hru, SOL_ALB(..).sol, LAT_SED.hru. The sensitivity analysis helps to identify major parameters within the watershed and their required precision for calibration (Ma et al., 2000). Of all the 21 parameters used, 12 are the most sensitive with less than 0.55 p-values and higher t stat values between 0 and 3 (Table 1). The sediment sensitive hydrological modelling parameters of the watershed starting with the most sensitive are ALPHA_BF.gw, CH_N2.rte, SOL_ALB(..).sol, OV_N. hru, SOL_AWC(..).sol, SURLAG.bsn, SOL_BD(..).sol, EPCO.hru, CN2.mgt, CH_K2.rte, SPCON.bsn, and GW_REVAP.gw.

**Table 1:** Relative sensitivity of sediment parameters to model output.

Parameter Name	t-Stat	P-Value
V__GW_REVAP.gw	0.55	0.58
V__SPCON.bsn	0.65	0.51
V_CH_K2.rte R__CN2.mgt	0.85 0.85	0.39 0.39
R__SOL_BD(..).sol	0.92	0.36
V__SURLAG.bsn	1.25	0.21
R__SOL_AWC(..).sol	1.38	0.17
V__EPCO.hru	0.87	0.38
R__OV_N.hru	1.84	0.07
V__SOL_ALB(..).sol	1.90	0.06
V__CH_N2.rte	2.19	0.03
V__ALPHA_BF.gw	2.59	0.01

Note that GW_REVAP.gw, is the groundwater “revap” coefficient. CH_K2.rte, effective hydraulic conductivity in main channel alluvium (mm/hr), SOL_AWC(.).sol, soil available water capacity, EPCO.hru, plant uptake compensation factor, SURLAG.bsn, surface runoff lag coefficient, OV_N, hru is manning's “n” value for the overland flow, SOL_ALB(..).sol, moist soil albedo (Mg/m3 or g/cm3), CN2.mgt is Initial SCS runoff curve number for moisture condition II, SPCON.bsn, the linear parameter for calculating the maximum amount of sediment that can be re-entrained during channel sediment routing, SPEXP.bsn, exponent parameter for calculating sediment re-entrained in channel sediment routing, SOL_BD(..).sol, moist bulk density (Mg/m3 or g/cm3) and ALPHA_BF, is base flow alpha factor.

The 12 sensitivity parameters in the watershed (Table 1), corroborate Schuol et al. (2007) and Adeogun et al. (2015) sensitivity analysis of West Africa and Nigeria respectively. These results laid a good foundation for the subsequent calibration and validation analysis that strongly rely on the sensitivity analysis for better model calibration and validation processes. The 12 parameters above can also serve as a base upon which further work can be carried out at the watershed scale in northern Nigeria since the application of the model is still relatively new in the nation.

### 3.2 Performance evaluation, calibration and validation of sediment yield

The similarity in climatic, soils, and land use conditions permits spatial calibration and validation method adopted in the watershed. Basins 69 and 83 observed monthly flow (2015–2017) was used for calibration and validated with another set of independent observed monthly flow (2015–2017) of Basins 62 and 79.

The streamflow validation parameters were used as a base to sediment yield calibration and validation. The model result was calibrated from March to October 2018 using the suspended sediment concentration of the four reaches (Kaduna, Sarkinpawa, Dinya and Gutalu) with the corresponding weather and streamflow data to the period for simulation in SWAT. The sediments spatial calibration and validation were carried out, using the observed sediment data collected from Basins 69 and 83 (March to October 2018) for calibration and those from Basins 62 and 79 (March to October 2018) for validation (Table 2).

**Table 2:** Sediment calibration & validation summary.

Sampling points	Calibration				Sampling points	Validation			
	NS	r^2^	p-factor	r-factor		NS	r^2^	p-factor	r-factor
Sarkinpawa (69)	0.01	0.53	0.88	2.25	Gutalu (79)	−0.11	0.06	0.88	2.96
Dinya (83)	0.91	0.93	1.00	7.57	Kaduna (62)	0.47	0.82	0.63	1.49

**Fig. 2:**
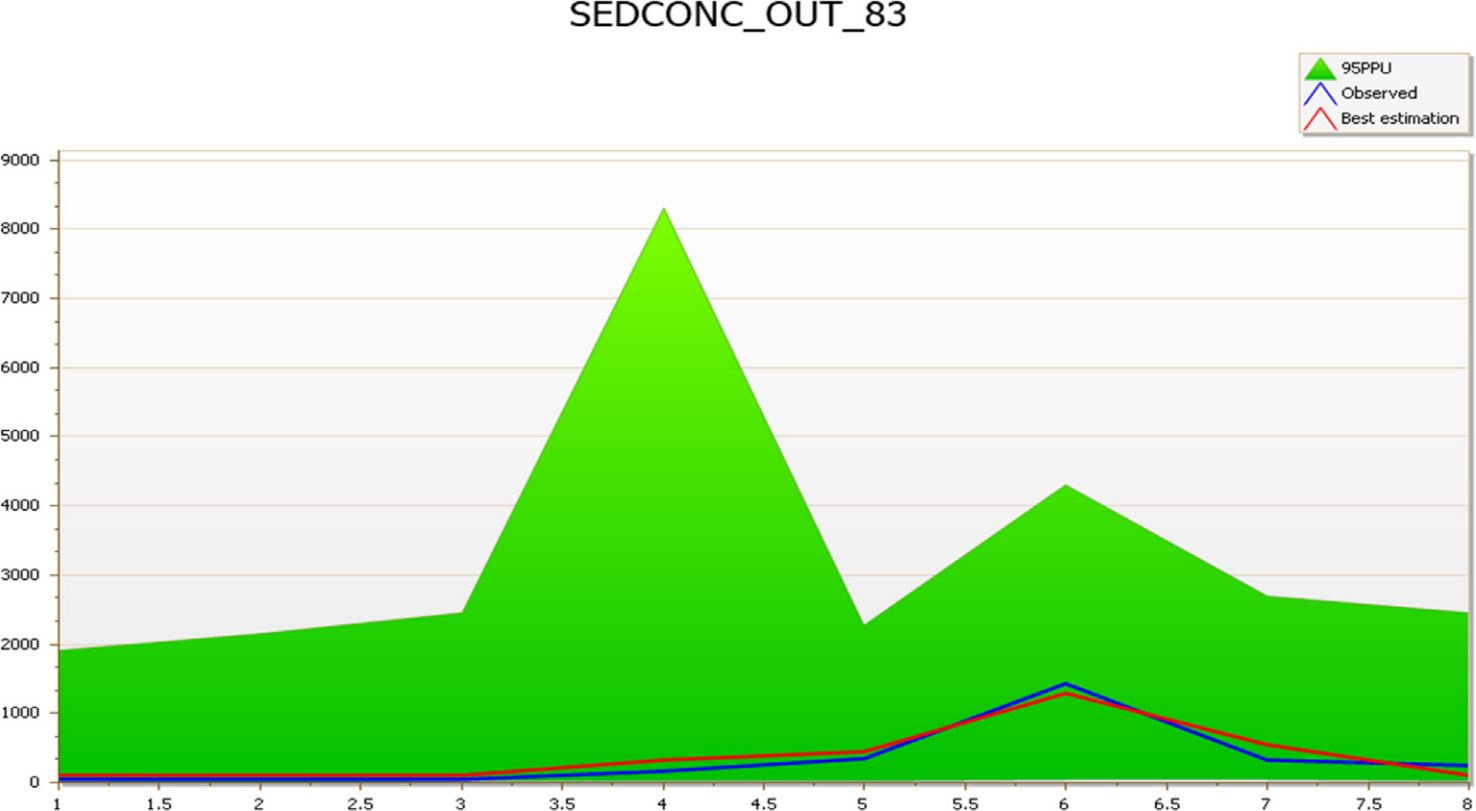
Sub-basin 83 comparison of simulated and observed sediment during the calibration period (March–October 2018).

Comparative analysis of the empirical and model prediction suggests a good correlation between the observed suspended sediment concentration and the simulated values in Basin 83 (Fig. 2). The model performance evaluation statistical summary in Basin 83 was highly satisfactory with NS of 0.91, r^2^ 93, p-factor of 1.00. These values strongly suggest perfect agreement between the observed and simulated values. However, the large r-factor of 7.57 is indicative of some level of uncertainty in the model. The result revealed a perfect correlation that could yield a very good validation result and good model predictions (Abbaspour et al., 2004, 2015; Krause et al., 2005; Abbaspour; 2015; van Liew and Garbrecht, 2003; Motovilov et al., 1999).

**Fig. 3:**
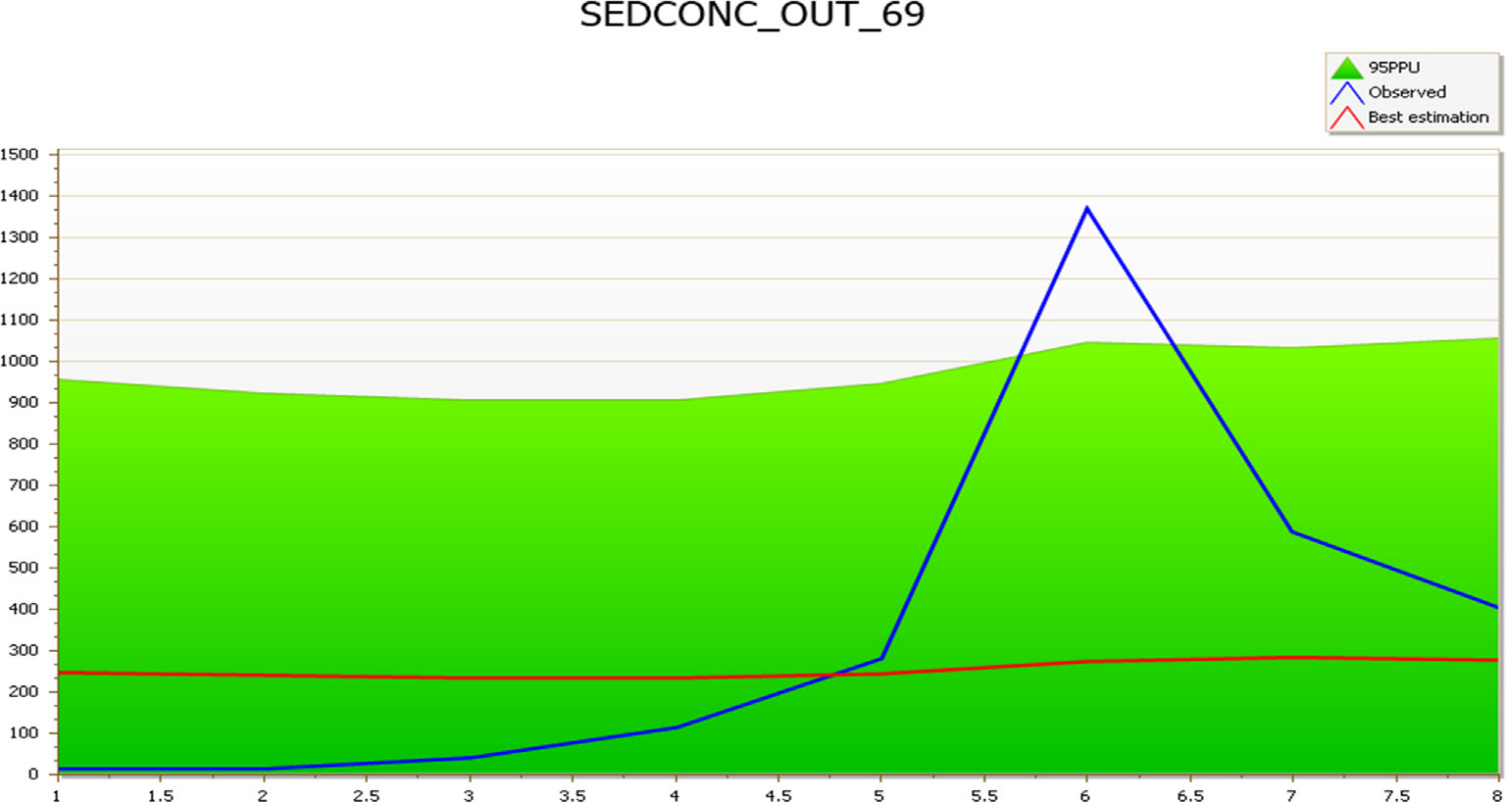
Sub-basin 69 comparison of simulated and observed sediment during the calibration period (March–October 2018).

The performance evaluation statistical summary in Basin 69 shows the coefficient of determination (r^2^) and p-factor values of 0.53 and of 0.88 respectively, thus capturing the flow dynamics, though with small NS (0.01) value indicative of mismatches in timing (Fig. 3). However, the r^2^ (0.53) and p-factor (0.88) yielded good simulations that captured the flow dynamics. These values according to Abbaspour et al. (2015) and Abbaspour (2015), are suggestive of good simulation.

However, r-factor of 2.25 in Basin 69 evidenced model uncertainties; which are activities that cannot be adequately captured by the model in the watershed. Model uncertainties connote those activities that may result from data errors, unassuming anthropogenic activities (e.g. mining) as well as users’ unknown model integrated processes. According to Abbaspour (2015), conceptual model uncertainty includes (i) processes occurring in the watershed that is not captured by the model (e.g. mining), (ii) processes included in the model, but their existence in the watershed is unknown to the model user (e.g. water transfer and irrigation etc), (iii) integrated processes in the SWAT model that are unknown to the user and (iv) processes not captured by the model and unknown to the model user (e.g. road construction dumping of waste and chemicals in the river etc.). The study area is marked by active artisanal mining (Fig. 6), a typical example of processes occurring in the watershed that is not captured by the model.

Usually, the process of mining especially when done by artisanal miners often leave heaps of tailings without reclamation, thus making it easier for runoff to transport such into nearby streams and rivers. Adeogun et al. (2015) also reported such a scenario at the riverbank of River Kontagora in Nigeria north central watershed.

**Fig. 4:**
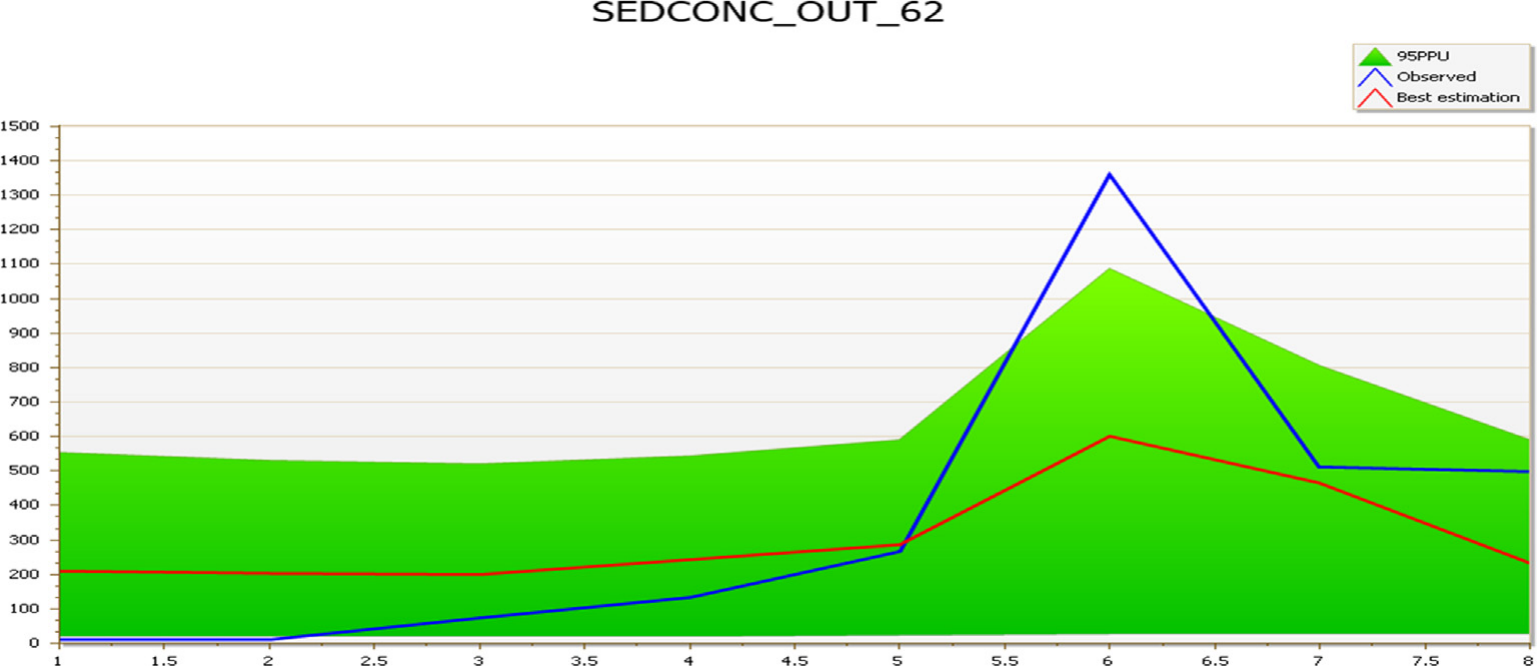
Sub-basin 62 comparison of simulated and observed sediment during the validation period (March–October).

The model validation was carried out using Kaduna and Gutalu reaches. The statistical summary for Basin 62 was good with NS of 0.47, r^2^ 0.82, and p-factor of 0.63 (Fig. 4). The basin result is good and could lead to good predictions going by existing literature (Abbaspour et al., 2015; van Liew and Garbrecht, 2003, Abbaspour et al., 2004, 2007).

**Fig. 5:**
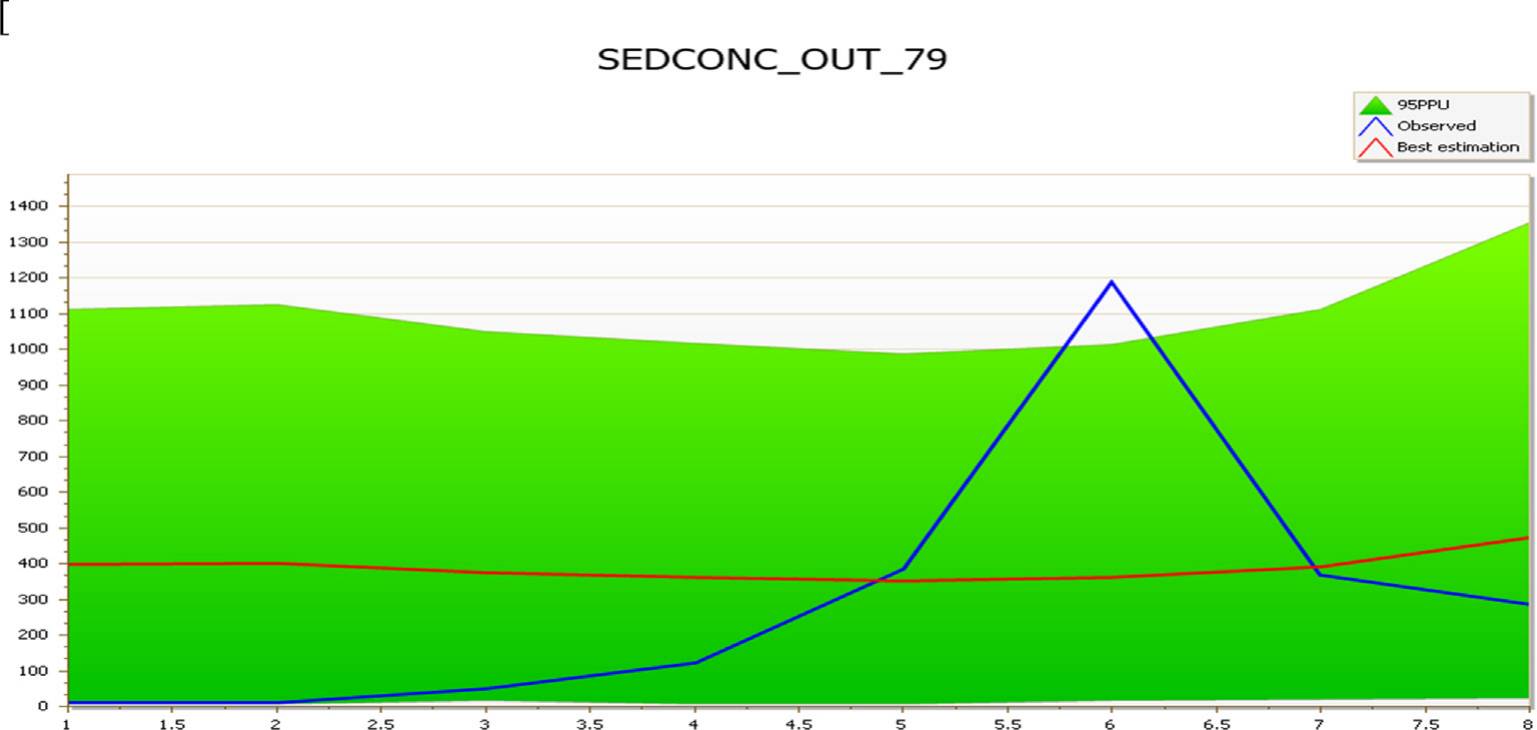
Sub-basin 79 comparison of simulated and observed sediment during the validation period (March–October 2018).

However, Basin 79 result was quite modest with NS of -0.11 and r^2^ of 0.06 and p-factor of 0.88 (Fig. 5). Abbaspour et al. (2015) reported a similar scenario in the Vistula river in Poland.

**Fig. 6:**
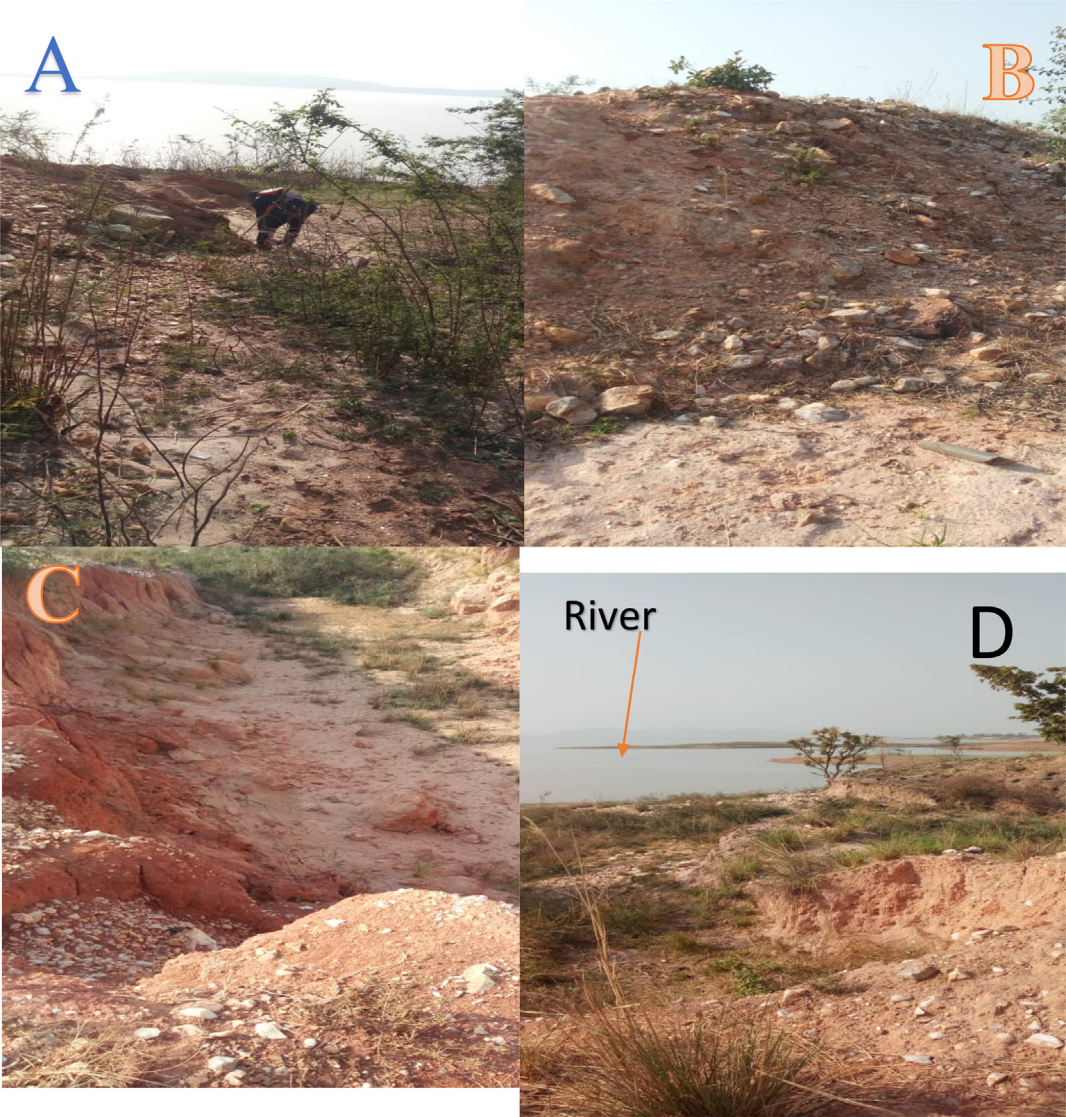
Local Miner soil excavation, B Tailings, C Residual of eroded soil and D eroded soil transported to Rivers Sarkinpawa and Gutalu.

The r-factor of 2.96 in Basin 79 can also be attributed to excess sediments load due to local miners’ activities that cannot be adequately captured by the model (Fig. 6). However, the mismatches in timing and activities that cannot be adequately captured by the model do not in any way affect the prediction since the r^2^ is within the acceptable standard. All these uncertainties according to Abbaspour et al. (2015) are expected to be experienced in a large watershed. A large watershed like Kaduna watershed will witnesses such uncertainties which explain the large r-factor predictions.

The overall results of the streamflow and sediment concentration based on the statistical evaluation performance using Nash-Sutcliff, Coefficient of Determination, p-factor and r-factor show that the SWAT model prediction in Kaduna watershed is under acceptable standards (Krause et al., 2005; Moriasi et al., 2007; Motovilov et al., 1999; Arnold et al., 2012a,b). Therefore, the model performance evaluation satisfied the prerequisite of SWAT model predictions. Hence, the model prediction is considered reliable going by the acceptable standard.

### 3.3 Sediment yield assessment and prediction of the study area watershed

**Fig. 7:**
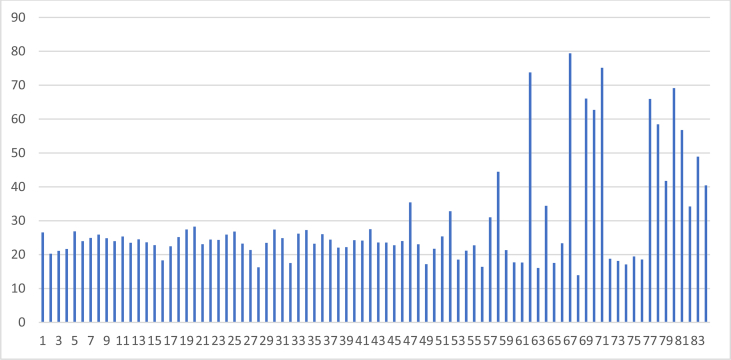
Simulated Annual Sediment Yield in each of the 84 Sub-basins in the Study Area.

The entire Kaduna watershed consists of 84 basins, with 69 in Kaduna sub-watershed, followed by Sarkinpawa and Gutalu with seven basins each and only one basin in Dinya (Fig. 1). Total predicted sediment yield of the 84 basins was presented (Fig. 7). The highest sediment yields were in basins 67, 71 (Sarkinpawa sub-watershed) and 62 (Kaduna sub-watershed) with sediment values of 79.4, 75.1and 73.8 t/h respectively. Lowest sediment yield was recorded in basins 28, 63 and 68 (Kaduna sub-watershed) with values of 16.3, 16.1 and 13.9 t/ha respectively.

**Fig. 8:**
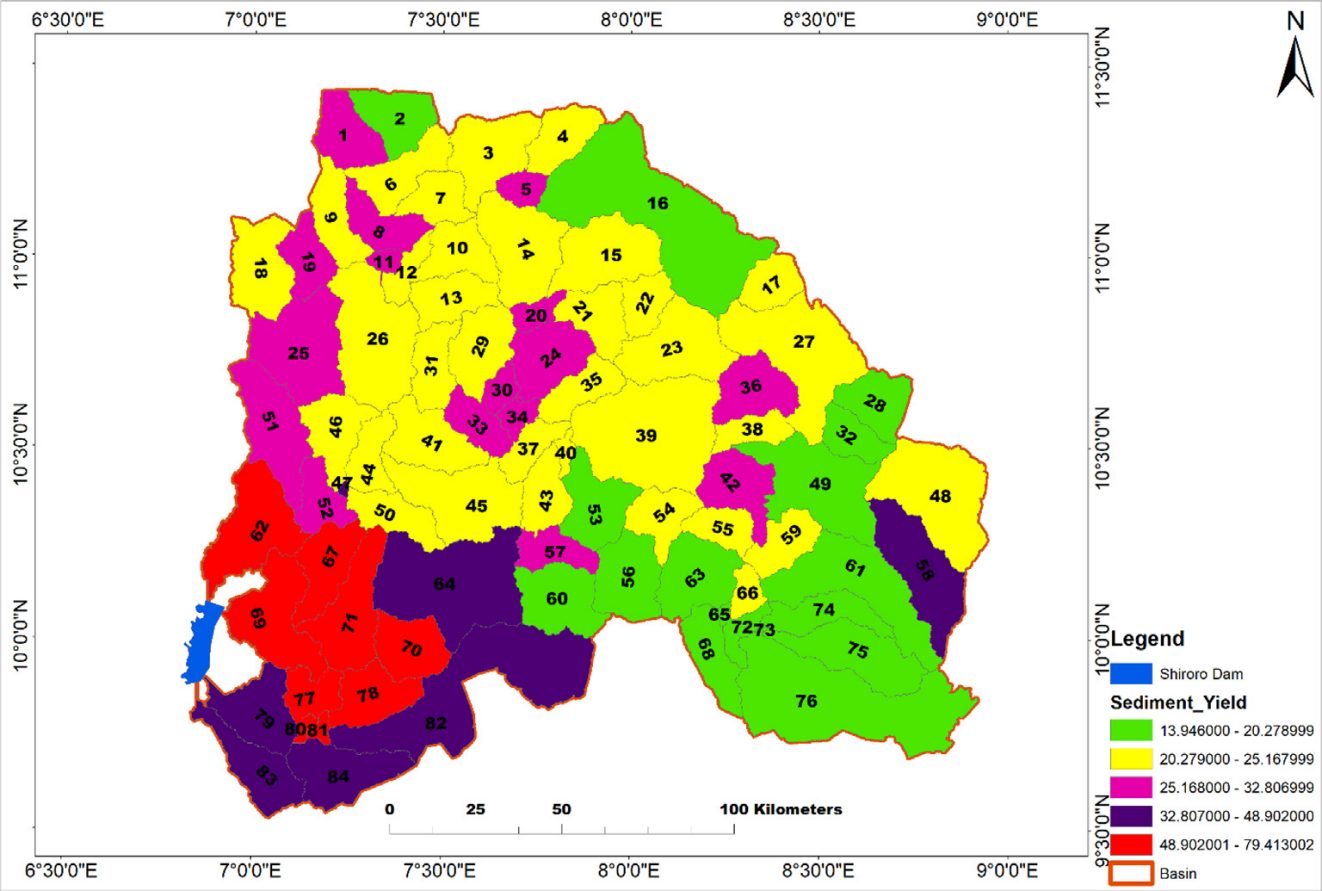
Sub-basins within Kaduna watershed showing sediment yield.

The model predicted surface runoff of ∼40.91 mm/yr, maximum upland sediment yield of 8.72 Mg/ha, average upland sediment yield of 0.98 Mg/ha and in-stream sediment change of -0.85 Mg/ha. Total sediment yield of about 2,438.358 t/ha in all the basins during the 32years simulation periods with three years warm-up period. The annual sediment yield in the watershed was estimated as 84.1t/ha/yr which translates to about 270 × 10^6^ tons of sediment between 1990 and 2018. Fig. 8 shows the watershed sediment yield categories based on five classes, and of these amounts, 68% came from Kaduna sub-watershed into the Shiroro reservoir; this is high enough to constitute possible threats to the sustainability of the Shiroro hydropower dam.

The predicted sediment yields of 84.1t/ha/yr (270 × 10^6^) tons is within the earlier predicted range in River Kaduna Nigeria by Abam (2001), who forecasted 96 t/km^2^/yr (66.66 × 10^6^) and Adeogun et al. (2018), who for 26 years predicted 255.8 t/ha/yr (8.31 × 10^9^) in Jebba (12,992 km^2^) upstream north central Nigeria. Given that rainfall is the dominant medium of sediment transport in the study area, earlier predicted value of 8.31 × 10^9^ appears to be outrageously high for a typical Guinea and Sudan climatic zones. Conversely, the 66.66 × 10^6^ quoted by Abam (2001) is also adjudged to be relatively too low for the study region. Betrie et al. (2011), predicted average sediment yield of 117×10^6^ tyr−^1^ for twelve (12) years simulations using SWAT model at the outlet of the upper Blue Nile in Ethiopia with almost similar climatic condition with Kaduna watershed. The result is close to NBCBN, (2005) prediction of 140×10^6^ tyr−^1^ (bed load inclusive) for the same area. Therefore, 270 × 10^6^ tons prediction can be considered good for 29 years simulations having in mind the climatic zone of the watershed (Guinea and Sudan) in Nigeria. Thus, the SWAT model application in sediment yield prediction in Kaduna watershed, Nigeria can be considered reliable.

Implementation of sustainable sediment management will prevent reservoir siltation and sedimentation (Anton et al., 2016; Kantoush and Sumi, 2010; Palmieri et al., 2001). Since, the main influence of reservoir sedimentation is a decrease in the life cycle of the reservoir (UNESCO, 2011; McCully, 1996; SedNet, 2006; Syvitsky et al., 2005; Ezugwu, 2013), the findings of this research are a potential tool to inform and guide stakeholders against the negative impacts of reservoir sedimentation. These, according to Yang (1996), and Patrick (1986) may involve (i) sediment gathering in front of power intakes that can lead to momentous costs for hydropower operations, (ii) blocking of the intake and bottom outlet structures (reservoir outlet works), (iii) abrasion of hydraulic machinery, thus declining generation efficiency and growing maintenance costs which could lead to expensive engineering resolutions. In worse cases, dredging is frequently required to remove excess sediment to permit a full flow of water through the intakes (UNESCO, 2011).

## 4 Conclusion

The results obtained from this study revealed that a properly calibrated SWAT model is appropriate for hydrology and sediment yield modeling at the watershed scale level in Nigeria. The predictions will help the dam managers to guide against negative impacts of reservoir sedimentation such as a reduction in a dam storage facility, sediment build-up in front of power intakes and bottom outlets which could lead to expensive engineering resolutions or reservoir dredging. Hence, extending the life of dams through careful management of sediment, using this kind of information should be a key priority. The application of these research findings will contribute to the prevention of possible greenhouse gasses generation from a household that would have been dependent on generators. Since, at full capacity operation, the dam will supply energy to about 404,000 households in north-central Nigeria. Hence, prevent further increase in the number of the current generator users in Nigeria estimated to be 60 million. Thus leading to a reduction in the amounts of carbon dioxide into the atmosphere in the region, which adds to the greenhouse effect, and believed to be raising earth's temperature. In all, calibrated and validated SWAT model is suitable as a management tool for planning and decision making at a watershed scale level in developing western Africa.

## Declarations

### Author contribution statement

Japheth Daramola: Conceived and designed the experiments; Performed the experiments; Contributed reagents, materials, analysis tools or data; Wrote the paper.

Toriman Mohd Ekhwan: Conceived and designed the experiments; Analyzed and interpreted the data; Contributed reagents, materials, analysis tools or data; Wrote the paper.

Jafaar Mokhtar, Kuok Choy Lam: Conceived and designed the experiments; Analyzed and interpreted the data; Wrote the paper.

Ganiyu Adeniyi Adeogun: Performed the experiments; Contributed reagents, materials, analysis tools or data.

### Funding statement

This research did not receive any specific grant from funding agencies in the public, commercial, or not-for-profit sectors.

### Competing interest statement

The authors declare no conflict of interest.

### Additional information

No additional information is available for this paper.
